# Multi-Omics Characterization of the 4T1 Murine Mammary Gland Tumor Model

**DOI:** 10.3389/fonc.2020.01195

**Published:** 2020-07-23

**Authors:** Barbara Schrörs, Sebastian Boegel, Christian Albrecht, Thomas Bukur, Valesca Bukur, Christoph Holtsträter, Christoph Ritzel, Katja Manninen, Arbel D. Tadmor, Mathias Vormehr, Ugur Sahin, Martin Löwer

**Affiliations:** ^1^TRON gGmbH - Translationale Onkologie an der Universitätsmedizin der Johannes Gutenberg-Universität Mainz Gemeinnützige GmbH, Mainz, Germany; ^2^University Medical Center of the Johannes Gutenberg, University Mainz, Mainz, Germany; ^3^BioNTech SE, Mainz, Germany; ^4^HI-TRON - Helmholtz-Institut für Translationale Onkologie Mainz, Mainz, Germany

**Keywords:** immunotherapy, cancer models, computational immunology, triple negative breast cancer, 4T1 murine mammary gland tumor cell line

## Abstract

**Background:** Tumor models are critical for our understanding of cancer and the development of cancer therapeutics. The 4T1 murine mammary cancer cell line is one of the most widely used breast cancer models. Here, we present an integrated map of the genome, transcriptome, and immunome of 4T1.

**Results:** We found Trp53 (Tp53) and Pik3g to be mutated. Other frequently mutated genes in breast cancer, including Brca1 and Brca2, are not mutated. For cancer related genes, Nav3, Cenpf, Muc5Ac, Mpp7, Gas1, MageD2, Dusp1, Ros, Polr2a, Rragd, Ros1, and Hoxa9 are mutated. Markers for cell proliferation like Top2a, Birc5, and Mki67 are highly expressed, so are markers for metastasis like Msln, Ect2, and Plk1, which are known to be overexpressed in triple-negative breast cancer (TNBC). TNBC markers are, compared to a mammary gland control sample, lower (Esr1), comparably low (Erbb2), or not expressed at all (Pgr). We also found testis cancer antigen Pbk as well as colon/gastrointestinal cancer antigens Gpa33 and Epcam to be highly expressed. Major histocompatibility complex (MHC) class I is expressed, while MHC class II is not. We identified 505 single nucleotide variations (SNVs) and 20 insertions and deletions (indels). Neoantigens derived from 22 SNVs and one deletion elicited CD8^+^ or CD4^+^ T cell responses in IFNγ-ELISpot assays. Twelve high-confidence fusion genes were observed. We did not observe significant downregulation of mismatch repair (MMR) genes or SNVs/indels impairing their function, providing evidence for 6-thioguanine resistance. Effects of the integration of the murine mammary tumor virus were observed at the genome and transcriptome level.

**Conclusions:** 4T1 cells share substantial molecular features with human TNBC. As 4T1 is a common model for metastatic tumors, our data supports the rational design of mode-of-action studies for pre-clinical evaluation of targeted immunotherapies.

## Introduction

The translational value of pre-clinical cancer studies is dependent on the availability of model systems that mimic the situation in the patient. The murine mammary carcinoma cell line 4T1 is widely used as syngeneic tumor model for human breast cancer [e.g., (1–3)], a tumor entity with the world-wide highest incidence^1^. This cell line was originally derived from a subpopulation of a spontaneously arising mammary tumor of a mouse mammary tumor virus (MMTV) positive BALB/c mouse foster nursed on a C3H mother (BALB/BfC3H) (4, 5). 4T1 can easily be transplanted into the mammary gland and was already described as poorly immunogenic, highly tumorigenic, invasive, and spontaneously metastasizing to distant organs (6). Thus, the location of the primary tumor and its metastatic spreading closely resemble the clinical course in patients. Moreover, 4T1 cells are used to specifically investigate triple-negative breast cancer (TNBC) [e.g., (7–9)] lacking protein expression of estrogen receptor (ER), progesterone receptor (PgR), and epidermal growth factor receptor 2 (ErbB2) (10). This triple-negative phenotype is estimated for more than 17% of breast cancers that are annually diagnosed (11).

In spite of being such a widely used system, until now mainly phenotypic characteristics of 4T1 have been compared to human (triple-negative) breast cancer in the literature, while no comprehensive genomic, transcriptomic, and immunomic overview has been provided that would complement the evaluation of 4T1 as adequate breast cancer or even TNBC model. In our study, we examined the 4T1 cell line from a multi-omic point of view to complete the picture.

## Materials and Methods

### Samples

BALB/cJ mice (Charles River) were kept in accordance with legal and ethical policies on animal research. The animal study was reviewed and approved by the federal authorities of Rhineland-Palatinate, Germany and all mice were kept in accordance with federal and state policies on animal research at the University of Mainz and BioNTech SE. Germline BALB/cJ DNA was extracted from mouse tail. 4T1 WT cells were purchased from ATCC. Third and 4th passages of cells were used for tumor experiments.

### Data

ENCODE RNA Sequencing data of adult BALB/c mammary gland tissue for differential expression analysis against 4T1 expression profiles was downloaded from the UCSC Genome Browser (12) repository:

- URL: http://hgdownload.cse.ucsc.edu/goldenPath/mm9/encodeDCC/wgEncodeCshlLongRnaSeq
- Files:
  wgEncodeCshlLongRnaSeqMamgAdult8wksFastqRd1Rep1.fastq.tgz
  wgEncodeCshlLongRnaSeqMamgAdult8wksFastqRd1Rep2.fastq.tgz
  wgEncodeCshlLongRnaSeqMamgAdult8wksFastqRd2Rep1.fastq.tgz
  wgEncodeCshlLongRnaSeqMamgAdult8wksFastqRd2Rep2.fastq.tgz

Female BALB/c RNA-Seq data sets for the comparison of the MHC expression were described before (13) and are available in the European Nucleotide Archive (see Data Availability Statement).

### High-Throughput Sequencing and Read Alignment

Exome capture from 4T1 and BALB/cJ mice were sequenced in duplicate using the Agilent Sure-Select solution-based mouse protein coding exome capture assay. 4T1 oligo(dT)-isolated RNA for gene expression profiling was prepared in duplicate. Libraries were sequenced on an Illumina HiSeq2500 (2 × 50 nt). DNA-derived sequence reads were aligned to the mm9 genome using bwa [(14); default options, version 0.5.9_r16]. Ambiguous reads mapping to multiple locations of the genome were removed. RNA-derived sequence reads were aligned to the mm9 genome using STAR [(15); default options, version 2.1.4a]. The sequencing reads are available in the European Nucleotide Archive (see Data Availability Statement).

### Mutation Detection

Somatic SNV and short insertion/deletion (indel) calling was performed using Strelka [(16); default options for whole exome sequencing, version 2.0.14] on each cell line or normal library replicate pair individually. The individual analysis runs resulted in 1,115 and 1,108 SNV candidates, with an overlap of 886 SNVs (66%) and in 60 and 58 indel candidates, with an overlap of 50 (74%).

### Transcriptome Profiling

Transcript abundance estimation was done with kallisto [(17); default options, version 0.42.4] on each cell line or normal sample library replicate individually using the mean transcripts per million (TPM) per transcript final value. Differential expression analysis was performed with edgeR [(18); default options, version 3.26.8] using the reported transcript counts of kallisto, summarized by adding up the counts of the respective transcripts associated with each gene. The TPM values of the technical replicates have a Pearson's correlation coefficient of more than 0.99. Enriched pathways (KEGG 2019 Mouse^2^) and gene ontologies (GO Biological Process 2018^3^) in differentially up- or downregulated genes were determined using Enrichr (19). The associated Enrichr libraries were used as background lists for comparison with enrichment analysis in TNBC subtypes (20).

Data from human TNBC studies (20–22) was obtained from the respective journal websites^4^,^5^,^6^. Data for mapping human and mouse gene symbols was obtained from the Jackson Laboratory^7^. TNBC and breast tissue short read data in fastq format was obtained from the short read archive (TNBC: accession number PRJNA607061, sample accession numbers are documented in Table S7).

TCGA BRCA expression values for ERBB2, ESR1, and PGR was obtained from the UCSC Xena browser (http://xena.ucsc.edu), using the “HTSeq FPKM-UQ” dataset. The clinical annotation including immunohistochemistry results was downloaded from the GDC Legacy site^8^. These tables were merged using the patient barcodes keeping only patients with non-missing and non-inconclusive results for the immunohistochemistry status of “Her2”, “Pr”, and “Esr”. This resulted in 808 data points. Principal component analysis was done in R with the “prcomp” function.

### Fusion Gene Detection

Fusion genes were detected with an in-house pipeline: We employed the “wisdom of crowds” approach (23), and applied four fusion detection tools, SOAPFuse, MapSplice2, InFusion and STARFusion (23–26) to two technical replicates of the 4T1 cell line. We used Ensembl GRCm38.95 as reference. SOAPFuse and STARFusion were run with default parameters, MapSplice2 was run with “–qual-scale phred33 –bam –seglen 20 –min-map-len 40” as additional parameters, and InFusion was run with “–skip-finished –min-unique-alignment-rate 0 –min-unique-split-reads 0 –allow-non-coding” as additional parameters. For run time improvement, we did a first manual pass of a STAR alignment to the mm10 reference genome and retained only non-matching and chimeric reads for further processing by the four fusion detection tools. In order to combine the eight resulting datasets (four tools applied to two replicates) we first created the union of results of all four tools for each replicate, followed by the intersection of both independent runs (one per replicate cell line RNA library). This was considered as high confidence result set.

### DNA Copy Number Calling

Absolute copy numbers were detected from exome capture data using Control-FREEC [(27), version 11.5]. Control-FREEC was run multiple times with different ploidy input parameters (ploidy = *x* for values of *x* = 2, 3, 4, or 5) on the merged alignment files (merged with the “merge” command from samtools). In addition, the following non-default parameters were set: forceGCcontentNormalization = 1, intercept = 0, minCNAlength = 3, sex = XX, step = 0, uniqueMatch = TRUE, contaminationAdjustment = FALSE.

The CNV calls were processed with custom Python and R scripts: The output segment copy numbers were assigned to gene symbols by intersection with gene coordinates. Using the gene symbols, the previously detected SNVs were mapped to the copy numbers. Computed variant allele frequencies (VAF) from read alignments were then compared to the expected allele frequency distribution based on discrete copy numbers. For e.g., for a copy number of 3 (as predicted by Control-FREEC), one would expect SNV VAFs in associated genes clustered around values of 0.33 (one allele mutated), 0.66 (two alleles mutated), and 1 (three alleles mutated). The best match was manually determined for a Control-FREEC ploidy value of 5.

### Transcript Assembly

RNA-Seq transcript assembly was done using trinity [(28); default options, version r20140413p1]. Assembled transcript contigs were mapped to human transcript sequences and the MMTV genome (GenBank accession number NC_001503.1) with blat (29).

### MHC Typing

MHC type of the 4T1 cells was determined from RNA-Seq reads as described in Castle et al. (13).

### MHC Expression

MHC expression was quantified using Sailfish [(30); default options, version 0.6.2] on an mm9 transcriptome index which represents C57BL/6 mice, combined with the expected BALB/cJ MHC sequences.

### Mutation Signatures

Mutation signatures (31) were computed with the R package YAPSA (default settings, version 1.4.0).

### Expression Profiling of Viral Genes

Virus genomes were downloaded in FASTA format from the NCBI Virus Genomes resource (32). Sequence reads were aligned using STAR [(15); version 2.5] to a combined reference genome containing murine genome sequences (mm9) and 7,807 virus genomes. We used a maximum mismatch ratio of 0.2, reporting ambiguous alignments only when the alignment scores matched the best alignment of the read.

For each of the virus accession numbers, the GenBank features “mRNA” and “CDS” were extracted from NCBI sources to create a virus gene database for expression analysis. Taxonomic information was extracted for filtering closely related viruses with lower read counts.

Viral gene expression was calculated using the built virus gene database and an in-house software as previously described (33). Any read overlapping a union model of all of a gene's isoforms was counted. All read counts were normalized to reads per kilobase of gene model per million mapped reads (RPKM) for all murine and viral genes.

### Neoantigen Selection for Immunogenicity Testing

The selection for the initial immunogenicity assessment was described earlier (34). For the subsequent testing of 11 additional 4T1-WT SNVs, the following more strict criteria were applied: (i) present in both replicates, (ii) hitting a transcript outside the untranslated region (UTR), (iii) resulting in a non-synonymous amino acid exchange (no stop gain or loss), (iv) mean expression in replicates > 0, (v) VAF in 4T1 DNA > 0, (vi) VAF in 4T1 RNA > 0.1, and (vii) VAF in RNA of an independent control mammary gland sample was 0. Indels were selected accordingly, but with a less stringent filter on the variant allele frequency in the tumor RNA (VAF_in_RNA > 0). Indels were subjected to confirmation via Sanger sequencing [performed as in (34)] which left two of the three pre-filtered indels for further experiments.

### Immunogenicity Testing

The immunogenicity assessment of SNV-derived neoantigens was performed as described earlier (34). For the testing of indel-derived mutated peptides, mice (*n* = 3) were vaccinated with repetitive intravenous injections of 40 μg RNA lipoplexes (35) on days 0, 7, and 14. Five days after the last immunization, splenocytes of mice were tested for recognition of 15-mer peptides spanning the complete mutated sequence (11 amino acid overlap). T-cell responses were measured via IFN-γ enzyme-linked immunospot assay (ELISpot) as previously described (34). In brief, 5 x 10^5^ splenocytes were stimulated overnight by addition of 2 μg/mL peptide at 37°C in anti-IFN-γ (10 μg/mL, clone AN18, Mabtech) coated Multiscreen 96-well plates (Millipore) and cytokine secretion was detected with an anti-IFN-γ antibody (1 μg/mL, clone R4-6A2, Mabtech). For subtyping of T-cell responses, CD8^+^ T cells were isolated from splenocytes via magnetic-bead based cell separation [Miltenyi Biotech, CD8a (Ly-2) MicroBeads] according to the manufacturer's recommendations. CD8^+^ T cell-depleted splenocytes served as a source for CD4^+^ T cells. 1.5 × 10^5^ isolated CD8^+^ T cells and 5 × 10^5^ cells derived from the CD4^+^ T cell containing flow-through were restimulated in an IFN-γ ELISpot as described above. 1 × 10^5^ syngeneic bone marrow derived-dendritic cells (34) served as antigen-presenting cells for CD8^+^ T cells.

## Results

### The 4T1 Tumor Genome

Using whole exome and RNA-Seq data, we assessed genomic variation patterns by comparing 4T1 to BALB/c DNA, examining copy number aberrations, indels, SNVs, and gene fusions. Moreover, we determined absolute DNA copy numbers.

**Figure 1:**
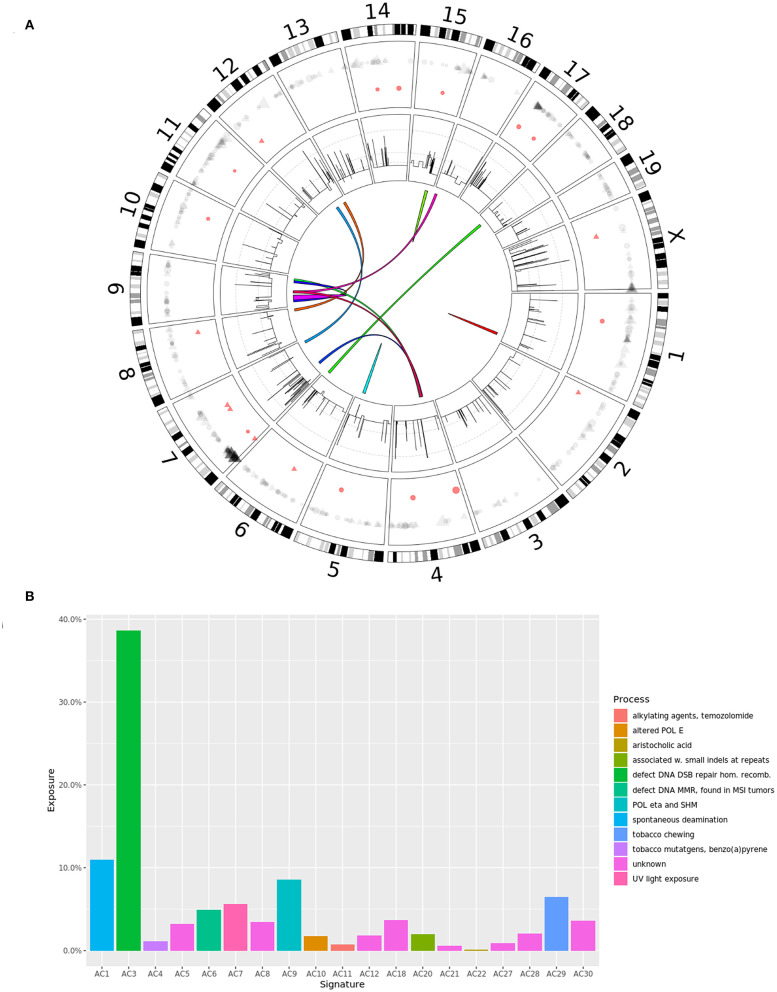
**(A)** Circos diagram showing the somatic alterations of the 4T1 cell line compared to wild type BALB/c mice: SNVs (outer circle, gray) and small indels (red), with point size scaled by variant allele frequency; CNVs (second circle from the outside), log scaled, with gray lines marking CN = 5, 10, and 50; fusion genes (middle). **(B)** Mutation signature of 4T1 somatic SNVs. Signatures with a computed exposure value of 0% are not shown.

No reads mapped to Y chromosome (DNA or RNA), which is expected as 4T1 originated from a female mouse. The analysis of the copy number profile revealed a median gene copy number of four, suggesting a tetraploid genome, although a sizable fraction of the genome seemed to be present in five copies (Figure 1A, second circle from the outside; Table S1). The findings were confirmed by a good agreement between the observed SNV allele frequencies and the allele frequency profile expected by the predicted gene copy number (e.g., for a copy number of four we expected SNV VAFs to be clustered around the values of 0.25, 0.5, 0.75, and 1). We observed known breast cancer oncogenes Akt1 and Sf3b1 (36) with focal amplifications (copy number six and seven, respectively), while pan-cancer oncogene Myc had a copy number of 11 (Table S1). Several known human tumor suppressor genes had a predicted copy number of less than four, with a possible functional impact (Table S1).

We identified 505 SNVs (Table S2, Figure 1A, outer circle, gray) and 20 short indels (Table S3, Figure 1A, outer circle, red) in transcripts, as well as 12 fusion events (Table S4, Figure 1A, middle). The majority of SNVs caused non-synonymous protein changes outside UTRs (264; 52%) including 248 missense and 16 non-sense variations (15 premature stops and one stop loss). Relative to the mouse genome (32 million protein-coding nucleotides), the 4T1 variation rate was 1.1 mutations per MB, which is within the range observed for human breast cancer (31). This number is an order of magnitude lower compared to the murine colon cancer model CT26, which suggests that CT26 is more likely to encode immunogenic epitopes than 4T1. The observed difference in the mutational load was in agreement with previous studies (37, 38), even though we detected a higher number of somatic mutations in both tumor models. We confirmed 45 of 47 (96%) and 193 of 246 (78%) previously reported SNVs in our data. Of the 264 non-synonymous SNVs, we found 91 (34%) mutations to be expressed (VAF > 0), which is comparable with a study in human TBNC that found ~36% of mutations to be expressed (39). We have recently shown a high correlation between the DNA and RNA mutation allele frequencies in three murine tumor models (including 4T1) (13). Here, using updated methods for transcript quantification and mutation calling, we were able to reproduce these results (*R*^2^ = 0.98, Figure S1), thus further corroborating that genes are equally transcribed from all alleles, mutated and wild-type (WT), in proportion to their DNA allele frequency.

Examining the mutational landscape in the 4T1 exome (Figure 1B), we found a higher prevalence of C>T, C>G, and C>A SNVs (Figure S2), which is in concordance with the somatic mutational signatures in human breast cancers (40). Interestingly, we found an overrepresentation of C>T transitions at XCG triplets (Figure S2; C is the mutated base, preceded by any nucleotide and followed by G), which is a known mutational mechanism due to deamination of methylated cytosines to thymine and has been observed in human breast cancers (41). C>T transitions showed the largest contribution to the mutational signatures in 4T1 and has been attributed to the activity of the APOBEC family of cytidine deaminases (42). Of note, Apobec3 has been found to provide partial protection in mice against infection with the oncogenic retrovirus MMTV (43), suggesting activation of this gene during MMTV infection and genome integration with subsequent cytosine deamination, resulting in the observed mutation pattern. The mutational signatures revealed a strong signal for signature AC3 (Figure 1B), which is associated with breast cancer and colloquially called “BRCAness,” followed by signature AC1, which is associated with spontaneous deamination. In contrast, signature AC2 was not found at all (and therefore not shown in Figure 1B), which would further strengthen the potential connection to APOBEC cytidine deaminases, as described above.

Of the most frequently mutated genes recently identified in breast cancer in general (41) and TNBC in particular (39) (Tp53, Pik3ca, Myc, Ccnd1, Pten, Erbb2, Znf703/Fgfr1 locus, Gata3, RB1, and Map3k1, Egfr), we only identified mutations in Trp53 (frameshift insertion of “A”) and Pik3cg (synonymous SNV) which is the catalytic subunit of class I PI3 kinases (similar to Pik3ca). In addition, we did not find mutations in breast cancer susceptibility genes Brca1 and Brca2. Further mutations in cancer-related genes included Nav3 (V1129L), Cenpf (D1327E), Muc5ac (A429P), Mpp7 (Q158R), Gas1 (G326R), Maged2 (A473S), Dusp1 (C24R), Ros1 (W1875C), Polr2a (M1102I), Rragd (L385P), and Hoxa9 (insertion of “G” in UTR). Variations in immune-relevant genes included Tlr8 (R613H), Tlr9 (N332K), and Lilrb3 (S91R).

Using RNA-Seq data of 4T1 replicates, we identified 12 fusion events (Table S4), including a fusion of Siva1 and Gas8, one regulating cell cycle progression/proliferation and apoptosis, the other being a putative tumor suppressor gene. None of them have been reported before in breast cancer (44, 45).

### MMTV Integration

MMTV is a milk-transmitted retrovirus that is oncogenic through integration into the host genome, thereby activating the expression of nearby genes (46). Multiple common insertion sites (CIS) have been identified and associated with candidate oncogenes and pathways involved in mammary tumorigenesis, including the Wnt and Fgf clusters (47, 48). A subset of CIS was significantly correlated with overexpression and deregulation of candidate oncogenes (49). We collected a set of 54 candidate genes for MMTV integration and compared their expression in 4T1 cells to that in normal mammary gland (Figure S3). About 68.5% of these genes showed significant down- or upregulation, while only about 42% of all genes of 4T1 cells were differentially expressed, suggesting MMTV integration as a possible cause. However, many pf the 54 candidate genes are involved in oncogenic pathways, so it is not clear if the observed differential expression are caused by the integration, potentially dysregulating a pathway or effect of the dysregulated pathway in the first place.

Moreover, we had direct evidence from RNA-Seq based transcriptome assembly of an integration site 5′ to the Fgfr2 gene (Figure S4). A CIS near Fgfr2 was associated with an increased copy number and overexpression of Fibroblast growth factor receptor 2 (Fgfr2) (47). While we just observed a copy number of four, three of eleven isoforms were significantly overexpressed in 4T1. Fgfr2 is a transmembrane tyrosine kinase receptor and its activation triggers a complex signal transduction network (via e.g., Ras-Raf-Mapk or Pik3-Akt pathway), which leads to transcription of genes involved in angiogenesis, cell migration, proliferation, differentiation and survival. There is evidence of deregulated activation of FGFR signaling in the pathogenesis of human cancers (46). FGFR2 amplifications have been found in 10% of gastric cancers (50) and were also found in a subset of human TNBC patients (39, 51); FGFR2 amplifications are estimated to occur in ~4% of TNBC samples, resulting in constitutive activation of FGFR2 (52). Increased expression of this gene is associated with poor overall survival and disease-free survival (53). This amplification is targetable with high sensitivity to FGFR inhibitors *in vitro* (52), an FGFR2-targeting antibody showed potent antitumor activity against human cancers in pre-clinical studies (54) and several FGFR tyrosine-kinase inhibitors are in clinical trials (54–56). However, the contribution of MMTV infection and initiation to human mammary carcinogenesis in general and FGFR2 amplification in particular is still highly debated (57). Of note, Notch4 and Krüppel-like factor 15 (Klf15) have been shown to be associated with MMTV CIS and although both genes are expressed in normal murine mammary gland, we do not find any isoform expressed in 4T1 possibly due to MMTV integration. Interestingly, while KLF15 has been recently proposed to be a tumor suppressor in breast cancer (58) and silencing this transcription factor results in a fitness advantage for the tumor, Notch-4 is a potent breast oncogene, overexpressed in TNBC (59) and Notch signaling is involved in mammary gland tumorigenesis (60).

### The 4T1 Transcriptome

Differential expression analysis of 4T1 cell RNA expression vs. healthy mammary gland tissue RNA revealed 12810 differentially expressed genes (FDR ≤ 5%, absolute log_2_ fold-change >1) out of 29,955 total genes in mm9 (Tables S5, S6). This set of differentially expressed genes is very similar to differentially expressed genes in human breast cancer: we compared the gene sets of two studies comparing TNBC epithelium to adjacent microdissected stroma (21) and TNBC to non-TNBC cancers (22). These studies allowed a gene set enrichment test, yielding *p*-values of 2.2 × 10^−16^ and 0.001002 (Fisher's exact test), respectively. Next, we compared pathways and gene ontologies (GO) that were significantly enriched (FDR ≤ 0.05, Table S7) in 4T1 differentially expressed genes to a study including different TNBC subtypes (20). Here, we only found significant overlap with top pathways and GO terms reported for subtype “Basal-like and immune suppressed (BLIS)” (*p*_*pathway*_ = 0.04506 and *p*_*GO*_ = 0.0142, Fisher's exact test). Furthermore, we analyzed RNA-Seq data of 57 TNBC breast cancer samples from the short read archive (PRJNA607061) and 66 breast tissue samples from the GTEx project (Table S8). All analysis steps were performed in analogy to the analysis of the 4T1 data. Here, we computed a *p*-value of 2.2 × 10^−16^ with Fisher's exact test when comparing the sets of differentially expressed genes. Moreover, the mean gene expression in TNBC is well-correlated to the gene expression in 4T1, as demonstrated by a Pearson's correlation coefficient of 0.727 (Figure S5).

**Figure 2:**
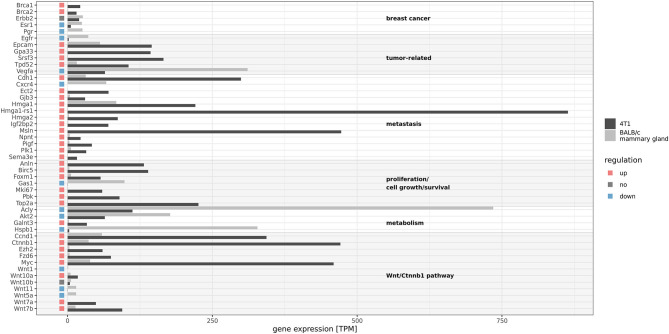
Gene expression of selected genes in 4T1 and BALB/c mammary gland. Gene expression was calculated as the sum of the determined transcript expression values in TPM (transcripts per million) and means of sample duplicates are given in the graph. Red and blue rectangles indicate differential expression (|log2FC| > 1, FDR ≤ 0.05). 4T1 exhibits characteristic gene expression patterns with respect to TNBC genes and other cancer- and metastasis-related genes.

Figure 2 shows the expression of a selection of relevant genes discussed below. The murine homologs of the typical genes associated with TNBC are Esr1, Pgr, and Erbb2. While Esr1 was about 2-fold downregulated and Pgr showed zero expression, Erbb2 had a comparable expression in 4T1 vs. the non-cancer mammary gland samples (about 20 TPM). However, compared to the ERBB2 expression in the TCGA human breast cancer (BRCA) cohort, this value was on the lower end of the expression level spectrum [not shown^9^ and (61)]. In order to investigate this detected mRNA expression, we compared the ERBB2, ESR1, and PGR mRNA expression in available TCGA breast cancer samples and grouped the expression values by the annotated result of the immunohistochemistry (IHC) assay. A principal component analysis (Figure S6) showed, that mRNA expression can separate IHC positives from negatives (albeit not perfectly). The data also showed that a negative IHC result is not necessarily associated with zero mRNA expression (Figure S7). With copy numbers of five, the three genes also did not divert form the general genomic copy number level. Moreover, genes Brca1 and Brca2 were highly overexpressed.

4T1 is a widely used model for metastatic breast cancer (62) and consistently, we found known metastasis-associated genes such as the differentiation antigen Msln (mesothelin), Cdh1, Sema3e, Gjb3, and Ect2 to be overexpressed. The latter one is known to be a key factor in progression of breast cancer (63) as well as in metastasis, and high expression is associated with poor prognosis for TNBC patients (64, 65). Overexpression of mesothelin was shown to promote invasion and metastasis in breast cancer cells (66). Interestingly, we found also High-mobility group protein HMG-I/HMG-Y (Hmga1) and Hmga-related sequence 1 (Hmga1-rs1) to be upregulated in 4T1 cells. Hmga1 is involved in promoting metastatic processes in breast cancer (67) and it has also been found to stimulate retroviral integration (68). Hmga2 is a driver of tumor metastasis (69) and Igf2bp2 is a downstream target gene (70). Both genes were highly expressed in 4T1 cells. In addition, we found a 6-fold overexpression of Nephronectin (Npnt) in 4T1 compared to the normal murine breast samples examined, in which we detected only weak signals of this gene (22.4 vs. 3.6 TPM). Npnt plays a role in kidney development, is associated with embryonal precursors of the urogenital system (71) as well as with integrin expression (72). High expression levels of Npnt have been observed in human thyroid (median: 277 TPM), human blood vessels (e.g., aorta, 200 TPM), human lung (161 TPM) and to a much lesser extent in human mammary tissue (14 TPM)^10^. Furthermore, Npnt has been suggested to have a role in promoting metastasation, as decreased expression in 4T1 tumors significantly inhibited spontaneous metastasis to the lung (73), further indicating the highly metastatic phenotype of 4T1. In contrast, we found an extremely low expression of Gas1, which plays a role in growth suppression. Also, growth factor Vegfa and growth factor receptor Egfr were downregulated.

Other deregulated genes are also described as being cancer-related, including Srsf3, which has a proto-oncogenic function and is frequently upregulated in various types of cancer (74). FOXM1 is a proto-oncogene involved in regulating the expression of genes that are specific for the G2/M DNA damage checkpoint during cell cycle prior to mitosis. Foxm1 has been found overexpressed in a variety of solid tumors, including breast cancer (75) and indeed, we also observed a 9-fold increase in 4T1 cells. PLK1 is also involved in the G2/M transition, found to be significantly overexpressed in TNBC and targeting this gene has been described as a potential therapeutic option for TNBC patients (76). Tumor protein D52 (Tpd52) was 6-fold upregulated, which is in consistence with reports showing high overexpression in many solid tumors and in particular breast cancer (77). Of note, we found the colon cancer antigen Gpa33 (78) to be highly expressed in 4T1 (143 TPM), not in normal murine breast (< 1 TPM) and not in any other human non-cancer tissue except colon (median: 111 TPM) and small intestine (median: 75 TPM) (data from^11^).

Among factors associated with a poor prognosis, proliferation markers Top2a, Mki67, and Birc5 (79–81) were highly expressed in 4T1, while almost absent in normal murine breast tissues. Pbk is also considered a marker for cellular proliferation (82) and is associated with poorer prognosis in lung cancer (83). Anln is highly expressed in breast cancer tissues (84) and a marker of poor prognosis in breast cancer (85) and indeed, we also found high expression of this gene in 4T1 (131.8 TPM). In addition, Pigf, which has been shown to enhance breast cancer motility (86) was overexpressed in 4T1 (42 TPM vs. 32.7 TPM). Genes related to metabolic regulation, such as Acly and Akt2, were downregulated. Polypeptide N-acetylgalactosaminyltransferase 3 (Galnt3) was upregulated in 4T1 and overexpression of this gene is associated with shorter progression-free survival in advanced ovarian cancer (87).

Moreover, Wnt7a and Wnt7b were upregulated in 4T1 cells, while other components of the Wnt/β-catenin pathway were downregulated (Wnt1, Wnt11, and Wnt5a). The role of Wnt10b in TNBC has been described before (88), indicating a direct effect on Hmga2 expression (see above). Furthermore, the gene Ezh2, known for its deregulatory activity of the Wnt pathway, was upregulated as well. Consequently, we found the Wnt target genes including the proto-oncogene Myc and the genes Ctnnb1, Ccnd1, and Fzd6 (Frizzled) to be upregulated (89).

As reported before (90), we found expression of the Murine Leukemia Virus (MuLV) gene coding for gp70, as well as of genes of the Murine osteosarcoma virus (NC_001506.1) and (confirming the genomic findings on MMTV integration) of all MMTV genes (Table S9).

### 6-Thioguanine Resistance

Due to the resistance to 6-thioguanine (6-TG) treatment, metastatic 4T1 cells can be precisely quantified even in distant organs (6). The cytotoxicity of 6-TG is based on the conversion of 6-TG into 2′-deoxy-6-thioguanosine triphosphate which can be incorporated into DNA (91). Deficiency in MMR, which is found in various cancer types (92), is associated with resistance to 6-TG (91). In 4T1, we did observe significant downregulation of Pold4 only, but none of the other MMR genes (Exo1, Lig1, Mlh1, Mlh3, Msh2, Msh3, Msh6, Pcna, Pcna-ps2, Pms2, Pold1, Pold2, Pold3, Rfc1, Rfc2, Rfc3, Rfc4, Rfc5, Rpa1, Rpa2, Rpa3, and Ssbp1; MSigDB: C2 curated gene sets, KEGG_MISMATCH_REPAIR, mouse orthologs obtained from^12^) at mRNA level (Table S6). Moreover, no non-synonymous SNVs or indels were detected in these genes, which might have impaired their function. In addition, mutational signatures AC6 and AC20 (associated with defective MMR) are present, but with relatively weak signals of about 5% and less (Figure 1B). Signatures AC15 and AC26 (also associated with defective MMR) are not detected. Diouf et al. (93) observed in human leukemia cells that MMR deficiency and thus an increased resistance to thiopurines can also result from a deregulated MSH2 degradation. While we again did not detect any mutations in the genes involved in regulating the stability of MSH2 (Mtor, Herc1, Prkcz, and Pik3c2b), we found Pik3c2b to be downregulated (Table S6). As the knockdown of PIK3C2B in human leukemia CCRF-CEM cells decreased sensitivity to 6-TG in comparison to control (93), lacking or reduced expression of Pik3c2b mRNA in 4T1 might explain the resistance to 6-TG treatment.

### MHC Expression

**Figure 3:**
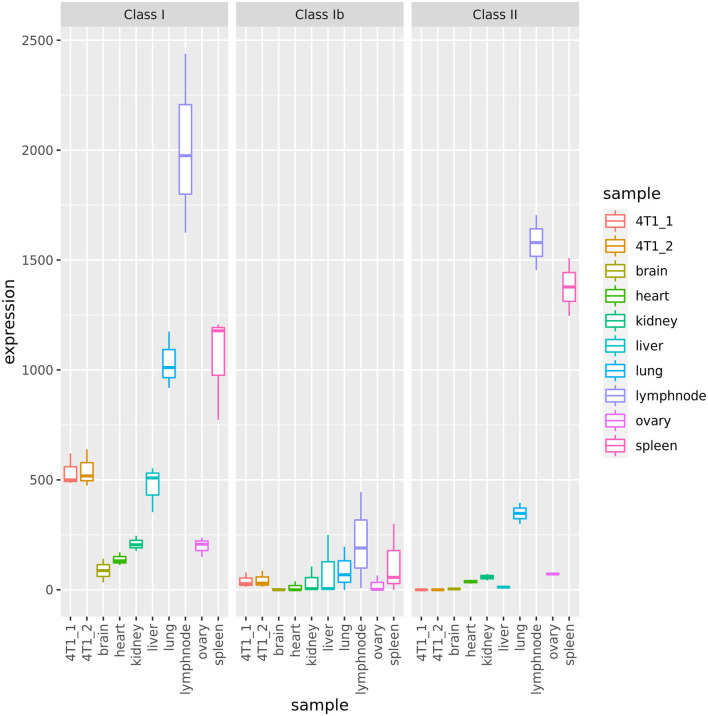
Expression of MHC genes in 4T1 cells and Balb/C tissue samples. 4T1_1 and 4T1_2 indicate the duplicate 4T1 RNA-Seq libraries.

The key players of the mammalian adaptive immune system are the major histocompatibility complex (MHC) molecules with the primary task to bind and present self, abnormal self, and foreign peptides derived from intracellular (MHC class I) or from extracellular proteins (MHC class II) on the surface of nucleated cells for recognition by T lymphocytes. Novel cancer immunotherapy concepts target tumor-specific antigens (either tumor-associated antigens or neo-epitopes) presented by MHC molecules of tumor cells. In general, non-cancer murine tissues show variable expression of MHC class I and class II, with lymphatic organs (i.e., lymph node, spleen) showing highest abundance of MHC transcripts and brain having the lowest MHC expression (Figure 3), which is in agreement with expression patterns of the human MHC system (94).

We confirmed that 4T1 cells have the same class I MHC haplotype as the parental BALB/c mice: H-2D^d^, H-2K^d^, and H-2L^d^. MHC class II could not be typed from RNA-Seq reads due to lack of expression. In 4T1, we found MHC class I and Ib loci to be expressed at comparable levels to normal (non-lymphatic) tissues (Figure 3, Table S10). In addition, β2-microglobulin (B2m), essential component of the MHC class I complex, and members of the MHC class I antigen presenting pathway were expressed (Figure S8). This suggests that MHC class I antigen presentation is functional and thus 4T1 cells are capable of presenting peptides and neo-epitopes to T effector cells. In contrast, 4T1 cells expressed neither MHC class II nor the MHC class II master regulator and transcriptional coactivator Ciita [Figure 3, Figure S8; (95)]. Both findings suggest that 4T1 cells do not have functional MHC class II antigen presentation.

### 4T1 Neoantigens

**Figure 4:**
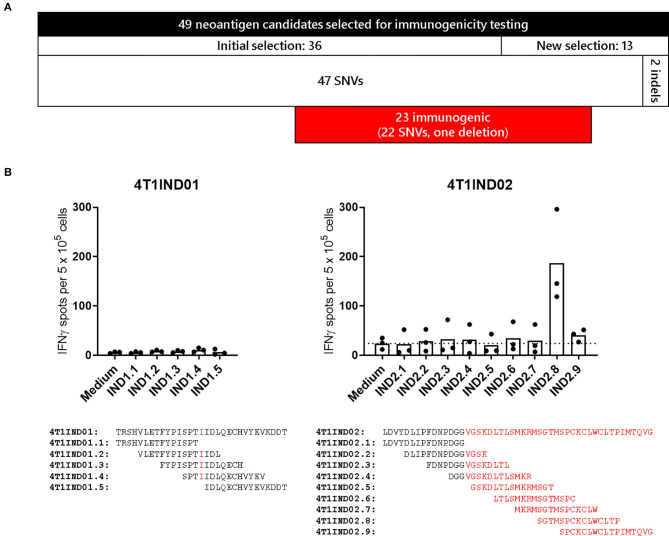
**(A)** Numerical overview of selected neoantigen candidates for immunogenicity testing. 22 out of 49 assessed targets were immunogenic. **(B)** Immunogenicity testing of indels. Splenocytes of immunized mice (*n* = 3 per indel) were tested 5 days after the last immunization via IFN-γ ELISpot for recognition of overlapping 15-mer peptides covering the complete 4T1IND01 and 4T1IND02 sequence as indicated below the graphs (11 amino acid overlap, new amino acids are highlighted in red). Columns indicate mean of spot counts. Peptide IND2.8 elicited IFN-γ spots >2-fold over background (dotted line, medium control).

To investigate the mutations with regard to their potential to elicit immune responses *in vivo*, experiments in mice were conducted. In a previous study (34), we already examined 38 SNVs detected in the 4T1-luc2-tdtomato mammary carcinoma (4T1-Luc) cell line. Thirty-six of these were also present in the WT 4T1 cell line, 16 of which were immunogenic. Based on the subsequent re-analysis of WT 4T1, we selected additional eleven SNVs and two indels for immunogenicity assessment (Figure 4A). This selection was done by filtering the available set of potential neoantigens in order to enrich for likely immunogenic peptide sequences (see Methods). To this end, a vaccine for each of the newly selected 13 mutations was engineered using antigen-encoding pharmacologically optimized lipoplexed RNA as vaccine format. As before, SNVs were flanked by 13 amino acids of WT sequence, in-frame indel mutations were flanked by 15 amino acids of WT sequence and frameshift mutations were investigated covering 15 WT amino acids upstream of the mutations as well as the whole sequence of new amino acids until reaching a stop codon. Mice (*n* = 3–5) were immunized intravenously three times within a 2-week timeframe. IFN-γ ELISpot of splenocytes stimulated with overlapping 15-mer peptides covering the respective vaccinated sequence was performed 5 days after the last immunization. With this, we found immune responses against additional six SNVs and one deletion (see Figure 4B for the results on the indels, Table S11 summarizes all immune responses). In total, we can thus report 22 SNVs and one deletion identified in 4T1 triggering immune responses in immunized mice. Of note, only four and 14 of these were derived from SNVs already reported before (37, 38). For a subset of 15 SNVs the WT counterpart was tested, which revealed that 10 responses were clearly specific for the mutated sequence. As already observed (34), most of the reactivities were elicited by CD4^+^ T cells (15 out of 21 analyzed mutations). Two SNVs were targeted by CD4^+^ and CD8^+^ T cells.

## Conclusion

The murine mammary cancer cell line 4T1 is one of the most often used model systems for breast cancer and in particular TNBC. Here, we could confirm that 4T1 indeed resembles metastatic TNBC at the transcriptional level with respect to key markers Esr1, Erbb2, and Pgr. In addition, compared to human TNBC data, we found good concordance on the level of differentially expressed genes and pathways and a reasonable correlation of raw expression values. The expression profile was in agreement with the metastatic phenotype of 4T1, as we found Msln, Ect2, and Plk1, and other genes associated to metastasis to be highly overexpressed in comparison to normal mammary gland. As described above, also a number of genes involved in proliferation and survival were deregulated. Moreover, it is known that the Wnt/β-catenin (Ctnnb1) pathway plays an important role in human breast cancers (96) with high activation rates and association with a poor prognosis (97). Some components of this pathway including Wnt target genes were upregulated in 4T1 cells. Overall, the observed profile reflected the complex interplay of various factors of tumorigenesis- and metastasis-driving signaling and allows for further mode-of-action investigation in the 4T1 tumor model.

On the mutation level, the raw numbers of mutations compare well against the CT26 colon cancer model. CT26 has 3,023 SNVs and 362 short indels, and in 4T1 we found an order of magnitude less variants (505 SNVs and 20 short indels). This is a similar relationship as observed for human colorectal and breast cancer (31) and supports previous findings (37, 38) as mentioned above. Differences in the absolute numbers in comparison to these reports might be due to genetic diversification of *in vitro* cell lines investigated at different laboratories at differing passage numbers (98) or different sequencing and mutation calling strategies.

Here and in a previous study (34), we determined *in vivo* immune responses against 22 SNVs (out of 49 tested, 45%) as well as one deletion (out of two indels tested) upon vaccination of BALB/c mice and 10 mutations (out of 15 immunogenic SNVs) showed mutation specificity. Although we did not examine all possible candidate neoantigens, the low mutational burden and the similarity to the basal-like and immune suppressed TNBC subtype suggest that 4T1 is a tumor model exhibiting relatively low immunogenicity. This is in agreement with others (37), while different studies argue the opposite, showing upregulation of many immune activation genes (38, 99) and thus immune cell infiltration in transplanted 4T1 tumors. Our 4T1 RNA-Seq data, however, was generated from the pure cell line. Accordingly, we could not see upregulation of immune-related genes. Nonetheless, 4T1 cells can secrete a plethora of inflammatory mediators and thereby modulate not only lymphocyte-mediated immune responses against the tumor, but also the innate microbial host defense (100–102). In future studies, the identified fusion transcripts might also be viable and interesting candidates for immunogenicity testing.

Besides the expression of MMTV at the RNA level and the deregulation of known genes with nearby insertion sites, we found direct evidence of MMTV integration near the gene Fgfr2. Combined with the relatively low mutational burden, we hypothesize that the MMTV infection and integration is the major genomic change causing eventually the TNBC-like phenotype. Interestingly, despite no observed somatic mutations in Brca1 or Brca2, a “BRCAness” mutation signature could be found (Figure 1B, signature AC3).

A very recent publication (38) underlined the importance of profiling tumor models to appropriately translate pre-clinical findings. The here presented genome, transcriptome, and immunome data serves as a baseline for further studies, examining e.g., tumor-host interactions in terms of immunogenicity and TNBC in general. Although the data sources are highly heterogeneous (resulting from different studies and sequencing experiments), a distinct overlap between our qualitative and quantitative findings and studies on human TNBC can be found and confirms our approach. Together, our study supports the rational design of pre-clinical studies with an important and established tumor model.

## Data Availability Statement

The datasets generated for this study can be found in the European Nucleotide Archive (https://www.ebi.ac.uk/ena/browser/home) with the study accession number PRJEB36287.

## Ethics Statement

This animal study was reviewed and approved by the federal authorities of Rhineland-Palatinate, Germany and all mice were kept in accordance with federal and state policies on animal research at the University of Mainz and BioNTech SE.

## Author Contributions

ML, BS, SB, and US contributed to the conception and design of the study. CA, VB, and KM were responsible for NGS sequencing. ML, BS, SB, TB, CH, and CR performed bioinformatics analyses. ML, BS, MV, and AT selected neoantigen candidates. MV planned and performed immunogenicity testing. ML, BS, and SB interpreted the data and wrote the first draft of the manuscript. CH, TB, and MV wrote sections of the manuscript. All authors contributed to manuscript revision, read, and approved the submitted version.

## Conflict of Interest

MV and US are employed by the company BioNTech SE. The remaining authors declare that the research was conducted in the absence of any commercial or financial relationships that could be construed as a potential conflict of interest.

## Footnotes

^1^http://gco.iarc.fr/today/data/factsheets/populations/900-world-fact-sheets.pdf

^2^https://amp.pharm.mssm.edu/Enrichr/geneSetLibrary?mode=text&libraryName=KEGG_2019_Mouse

^3^https://amp.pharm.mssm.edu/Enrichr/geneSetLibrary?mode=text&libraryName=GO_Biological_Process_2018

^4^https://www.ncbi.nlm.nih.gov/pmc/articles/PMC4911051/bin/pone.0157368.s007.xlsx

^5^http://downloads.hindawi.com/journals/bmri/2018/2760918.f1.docx

^6^https://static-content.springer.com/esm/art%3A10.1186%2Fs13058-016-0690-8/MediaObjects/13058_2016_690_MOESM1_ESM.docx

^7^http://www.informatics.jax.org/downloads/reports/HOM_MouseHumanSequence.rpt

^8^file URL: https://portal.gdc.cancer.gov/legacy-archive/files/735bc5ff-86d1-421a-8693-6e6f92055563

^9^http://gepia.cancer-pku.cn/detail.php?gene=ERBB2

^10^https://gtexportal.org/home/gene/NPNT (accessed January 9, 2020)

^11^https://gtexportal.org/home/gene/GPA33 (accessed January 9, 2020)

^12^http://bioinf.wehi.edu.au/software/MSigDB/

## Supplementary Material

Figure S1 Comparison of DNA and RNA variant allele frequency (VAF) in 4T1 cells. The Pearson correlation coefficient is 0.977.

Figure S2 Abundance of nucleotide substitutions in 4T1 cells with respect to nucleotide triplets.

Figure S3 Differential expression of MMTV integration effector genes. Colored dots indicate differential expression in 4T1 vs. BALB/c mammary gland. Red gene labels indicate genes that are described as upregulated in the literature.

Figure S4 Schematic view of proposed MMTV integration in Fgfr2 gene. Upper panel shows a UCSC Genome Browser view of an alignment of assembled sequence c75264_g4_i1 to the mm9 genome. The middle part shows the assembled sequence (blue) and the part mapping to Fgfr2 (red). Numbers indicate parts of the sequence mapping to Fgfr2 (red) and MMTV (green). The lower panel shows a schematic of Betaretrovirus genome, for which MMTV is a reference strain (taken from https://viralzone.expasy.org/66).

Figure S5 Mean gene expression of TNBC plotted against mean gene expression in orthologous genes of 4T1. Counts per million (cmp) were computed by edgeR.

Figure S6 Scatterplot of a principal component analysis of TCGA BRCA gene expression of genes ERBB2, ESR1, and PGR. Shown are the first two principal components (PC1 and PC2). Ellipses indicate normal-probability contours.

Figure S7 Boxplot of TCGA BRCA gene expression of genes ERBB2, ESR1, and PGR, separated by TNBC status. Expression on y-axis is given as log_2_ (FPKM+1) units.

Figure S8 Gene expression of members of the MHC class I and II antigen presenting pathway in 4T1 and BALB/c mammary gland.

Table S1 Raw Control-FREEC output (sheet 1) and predicted absolute gene copy numbers of 4T1 genes (sheet 2).

Table S2 Somatic SNVs in 4T1, including annotation on amino acid substitutions, affected genes/transcripts, expression of these, and coverage/VAF in the DNA/RNA NGS libraries.

Table S3 Somatic INDELs in 4T1, including annotation on frameshift, affected genes/transcripts and coverage/VAF in the DNA/RNA NGS libraries. A VAF of −1 means “not covered,” while a VAF of 0 indicates coverage but absence of the variant allele.

Table S4 Fusion genes in 4T1, including predicted positions of breakpoints, number of junction reads, and spanning read pairs and the program that detected a fusion.

Table S5 Gene expression in 4T1 and BALB/c mammary gland in TPM.

Table S6 Differential gene expression in 4T1 vs. BALB/c mammary gland, showing log fold change, FDR, and baseline expression values.

Table S7 Gene set and pathway enrichment in differentially expressed genes of 4T1 cells for upregulated and downregulated genes in GO gene sets and KEGG pathways, respectively (sheets are labeled “up GO”, “up KEGG”, “down GO”, and “down KEGG”, respectively).

Table S8 Differential gene expression in human TNBC vs. breast tissue, showing log fold change, FDR and baseline expression values.

Table S9 Expression in RPKM of MMTV genes for two replicates of 4T1 RNA-Seq libraries.

Table S10 Expression values in TPM of MHC genes in 4T1 and BALB/c tissues. The used reference sequences from the UCSC known genes or Genbank are also listed.

Table S11 Results of immunogenicity testing, including details on mutation, amino acid substitution, the result of the ELISpot assay, the subtype of the T-cell response, and the specificity when compared to a WT control.
