# Supplementary figures and images for: Multi-Omics Characterization of the 4T1 Murine Mammary Gland Tumor Model
Source: Front Oncol. 2020 Jul 23;10:1195. doi: 10.3389/fonc.2020.01195 (PMC7390911; doi:10.3389/fonc.2020.01195)

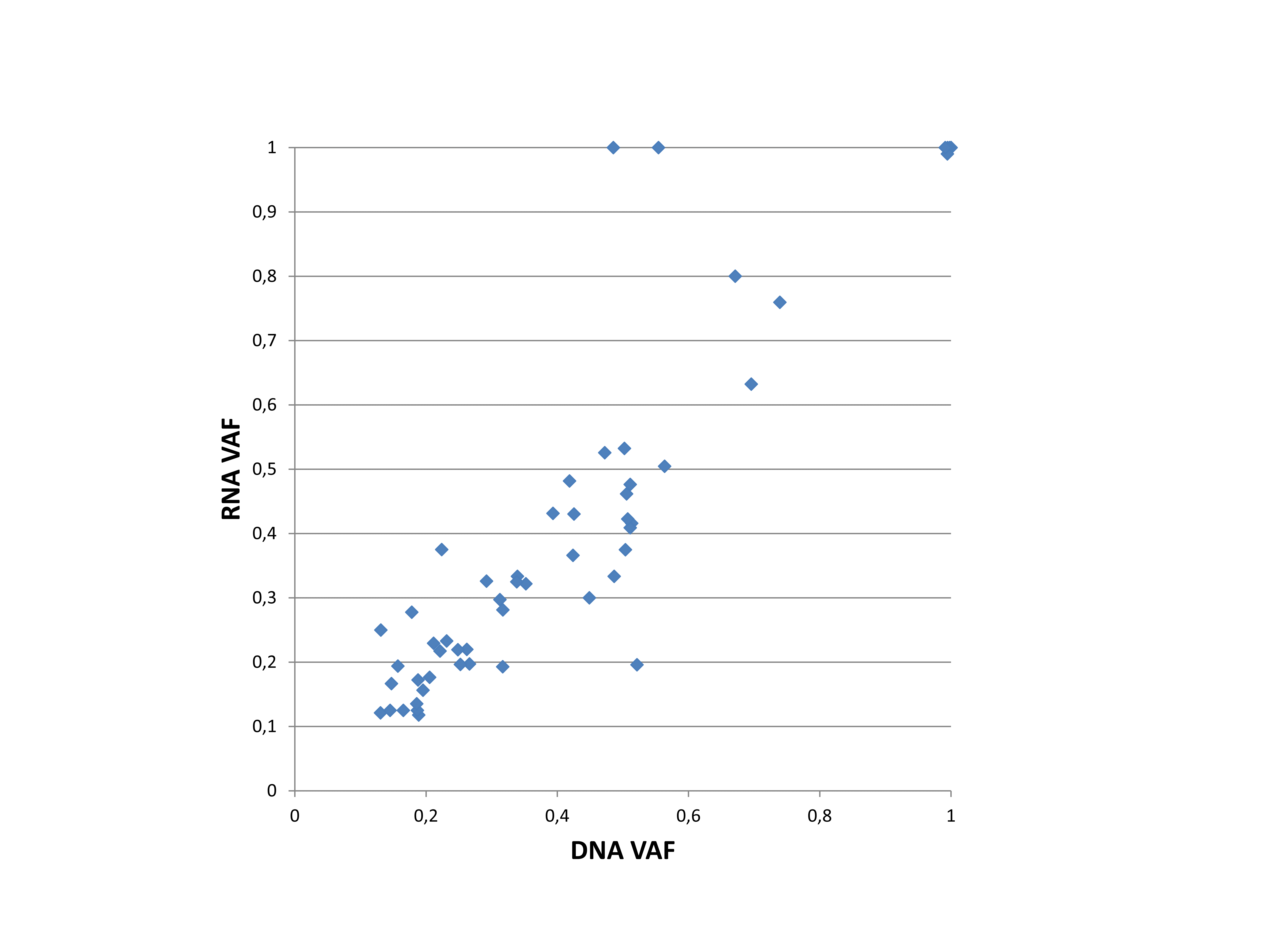

Supplement: Figure S1 — Comparison of DNA and RNA variant allele frequency (VAF) in 4T1 cells. The Pearson correlation coefficient is 0.977. [file Image_1.PNG]

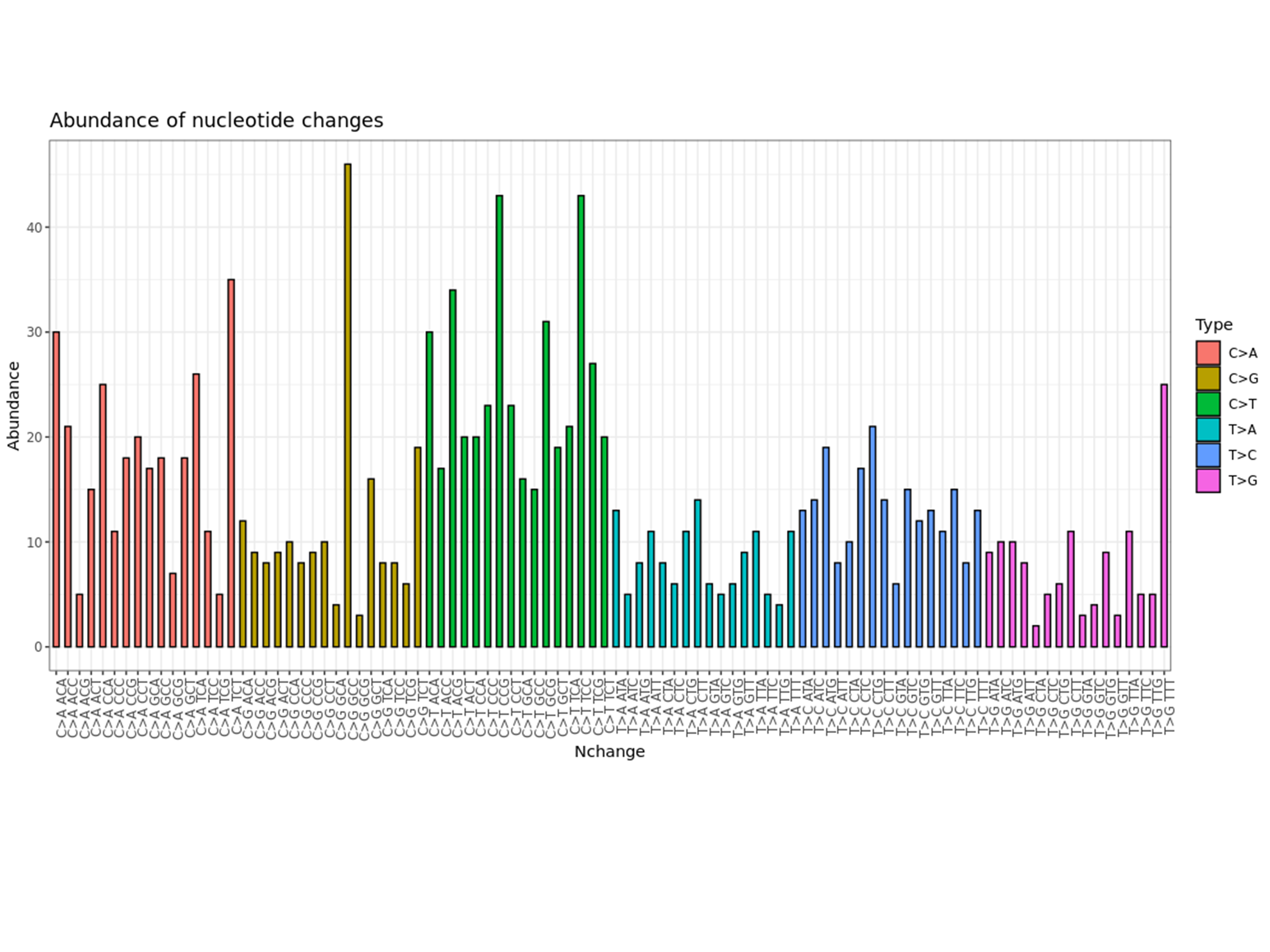

Supplement: Figure S2 — Abundance of nucleotide substitutions in 4T1 cells with respect to nucleotide triplets. [file Image_2.TIF]

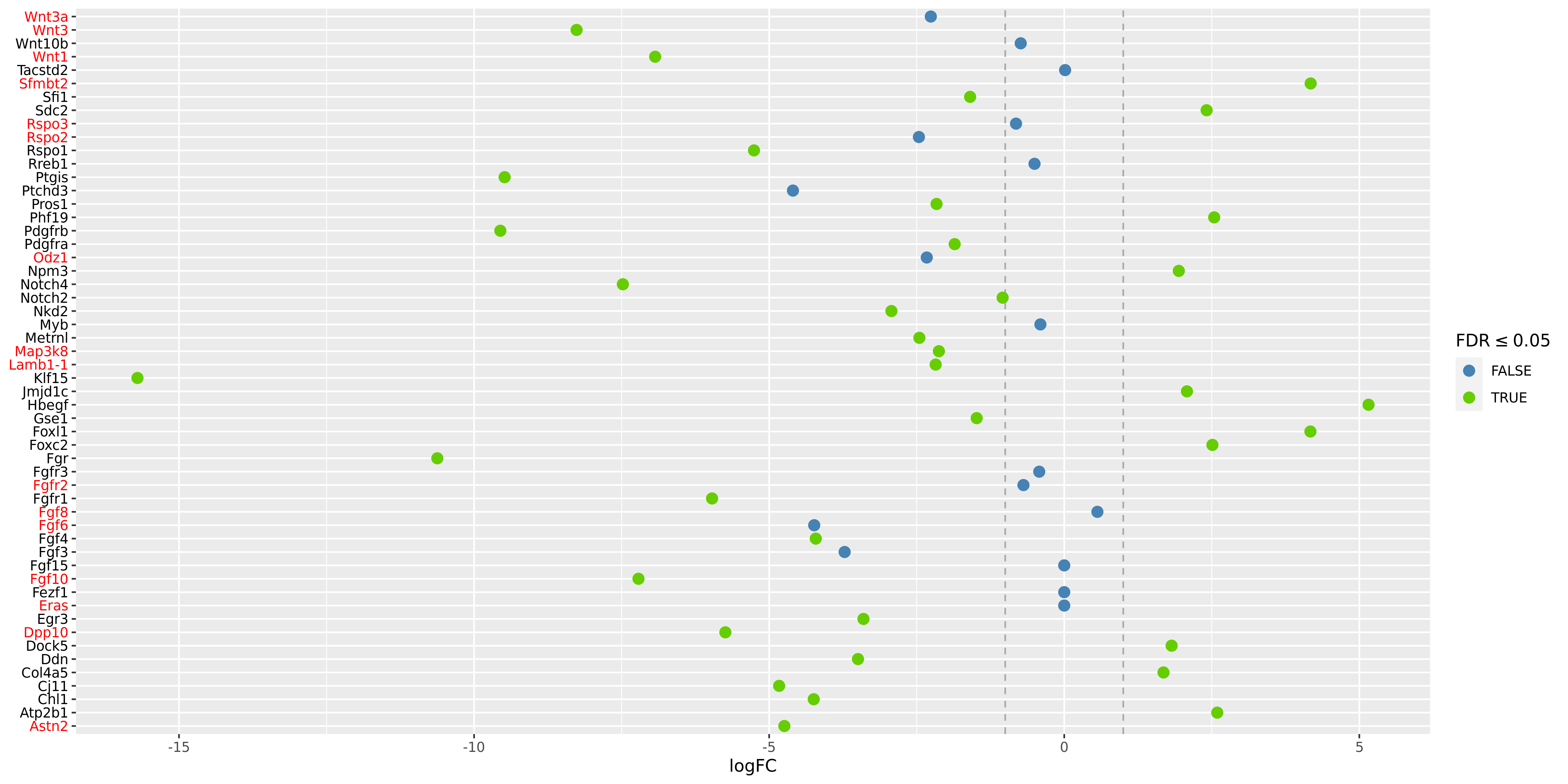

Supplement: Figure S3 — Differential expression of MMTV integration effector genes. Colored dots indicate differential expression in 4T1 vs. BALB/c mammary gland. Red gene labels indicate genes that are described as upregulated in the literature. [file Image_3.TIFF]

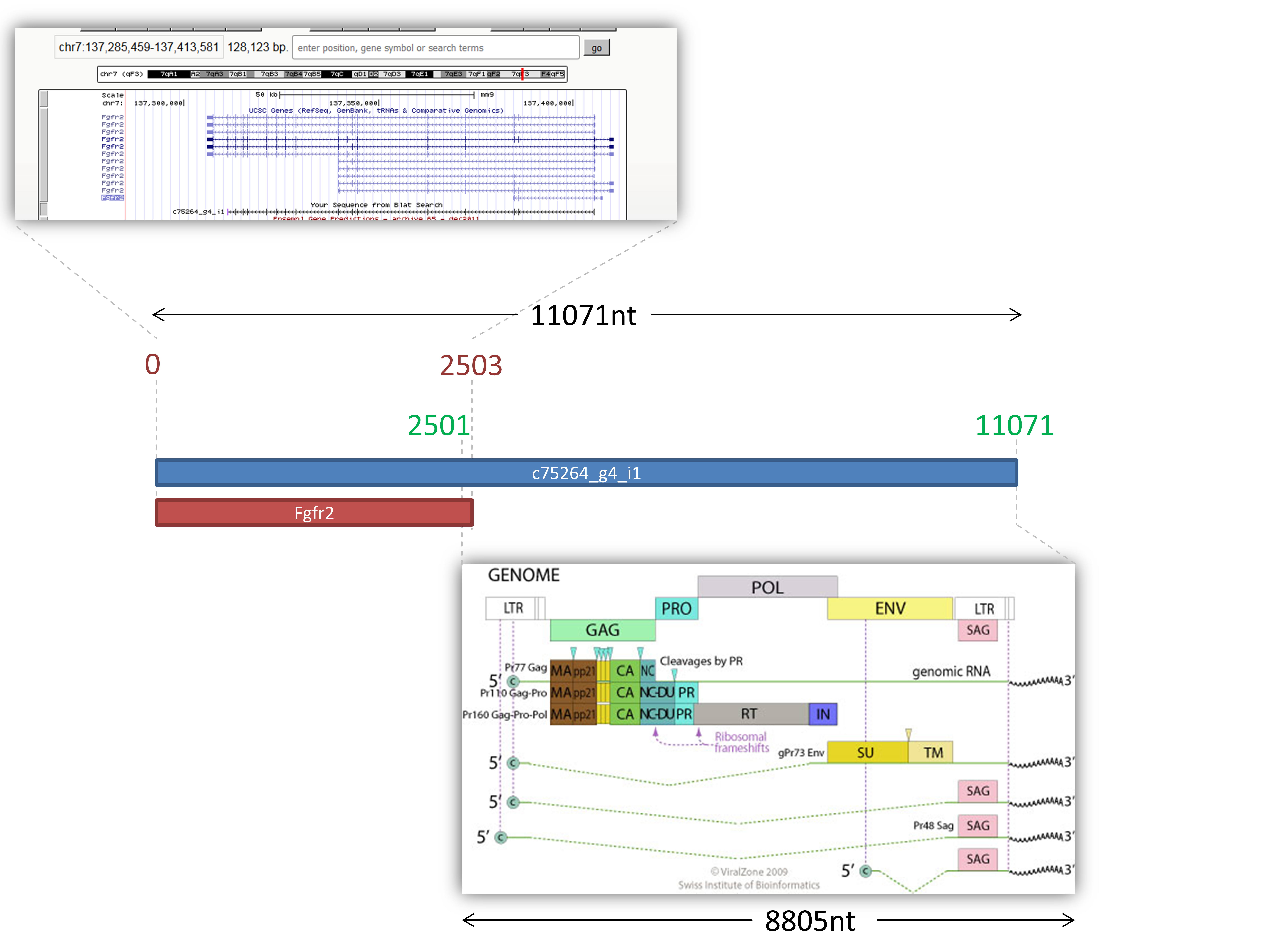

Supplement: Figure S4 — Schematic view of proposed MMTV integration in Fgfr2 gene. Upper panel shows a UCSC Genome Browser view of an alignment of assembled sequence c75264_g4_i1 to the mm9 genome. The middle part shows the assembled sequence (blue) and the part mapping to Fgfr2 (red). Numbers indicate parts of the sequence mapping to Fgfr2 (red) and MMTV (green). The lower panel shows a schematic of Betaretrovirus genome, for which MMTV is a reference strain (taken from https://viralzone.expasy.org/66). [file Image_4.TIF]

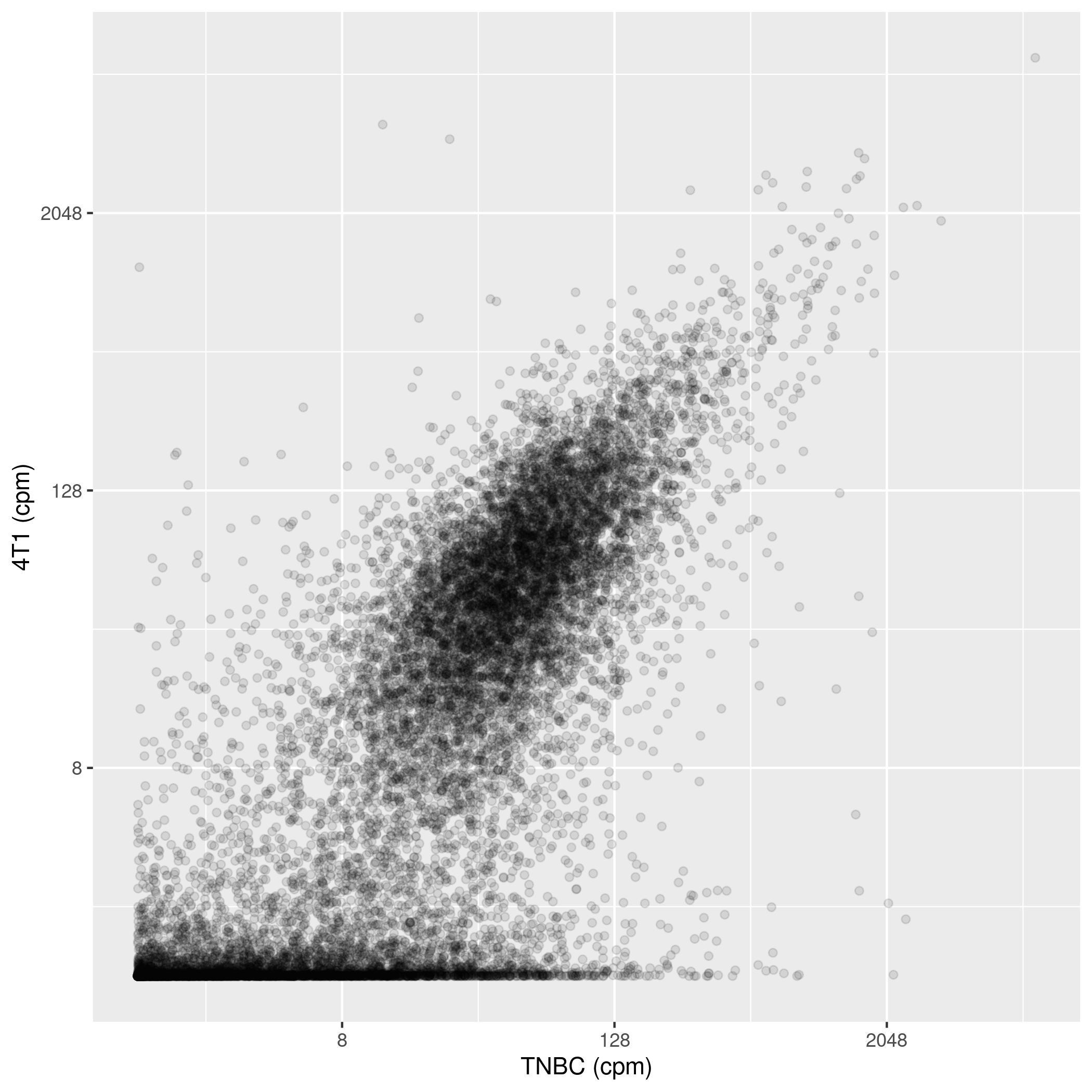

Supplement: Figure S5 — Mean gene expression of TNBC plotted against mean gene expression in orthologous genes of 4T1. Counts per million (cmp) were computed by edgeR. [file Image_5.TIFF]

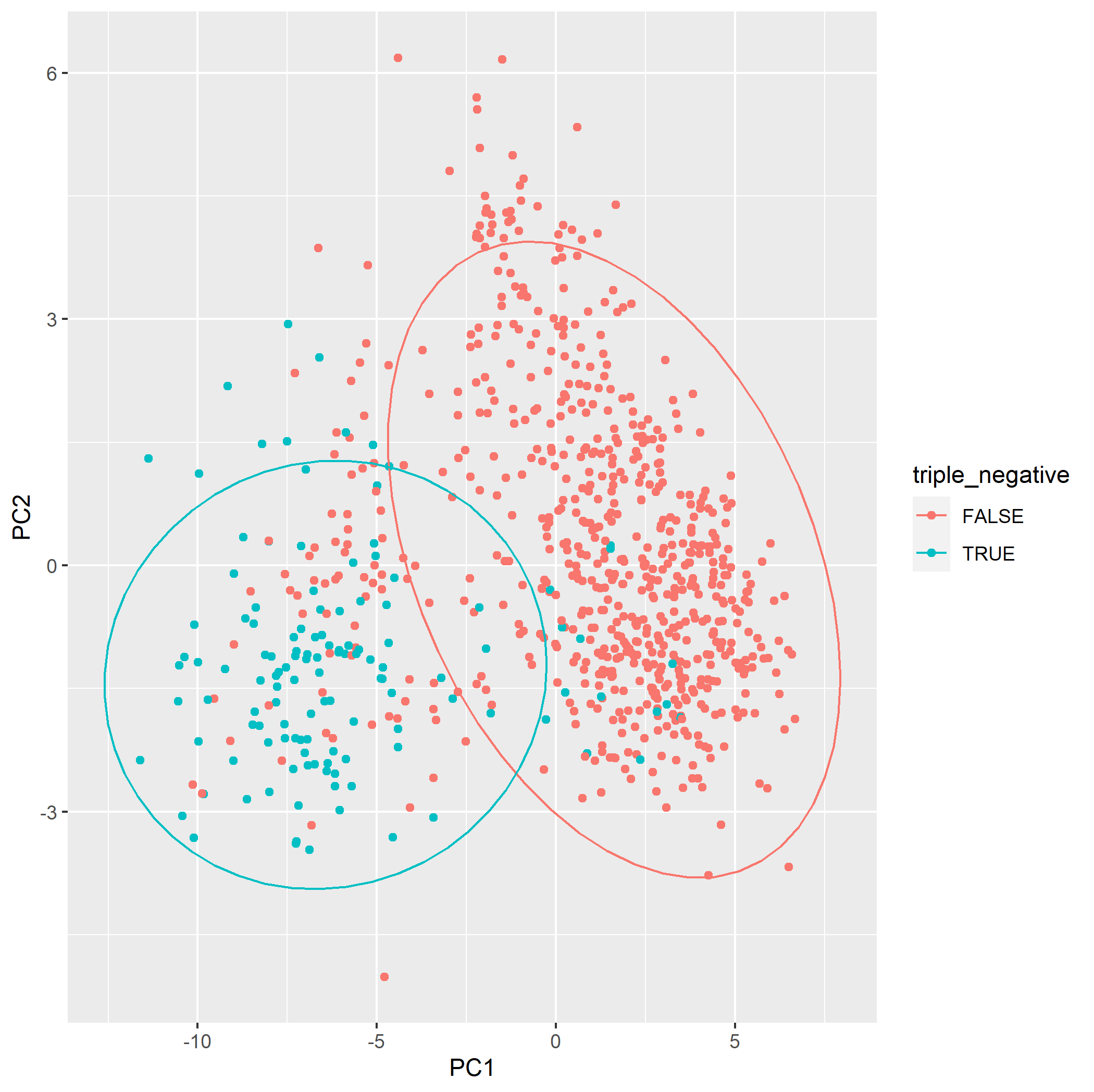

Supplement: Figure S6 — Scatterplot of a principal component analysis of TCGA BRCA gene expression of genes ERBB2, ESR1, and PGR. Shown are the first two principal components (PC1 and PC2). Ellipses indicate normal-probability contours. [file Image_6.PNG]

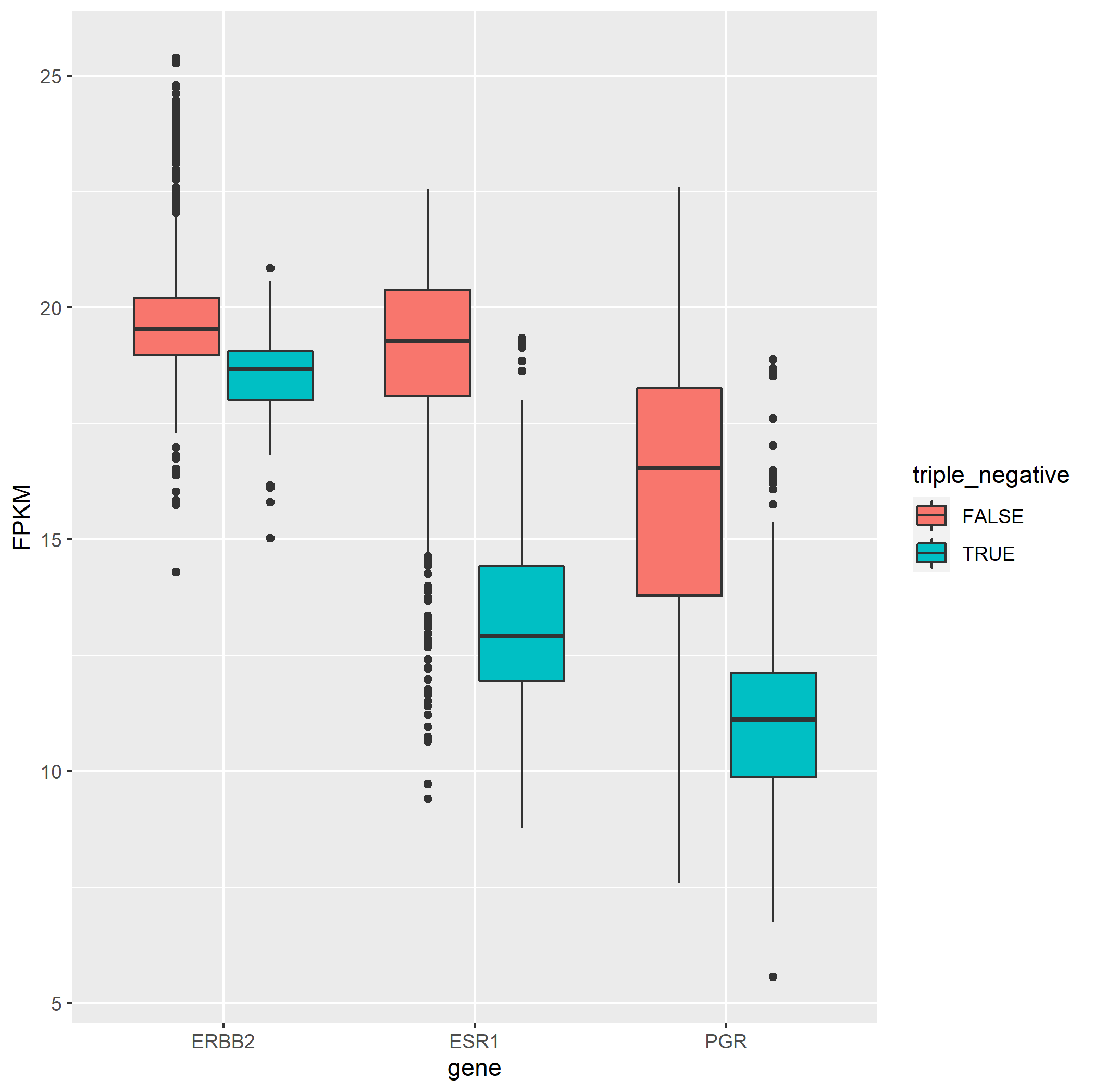

Supplement: Figure S7 — Boxplot of TCGA BRCA gene expression of genes ERBB2, ESR1, and PGR, separated by TNBC status. Expression on y-axis is given as log2 (FPKM+1) units. [file Image_7.PNG]

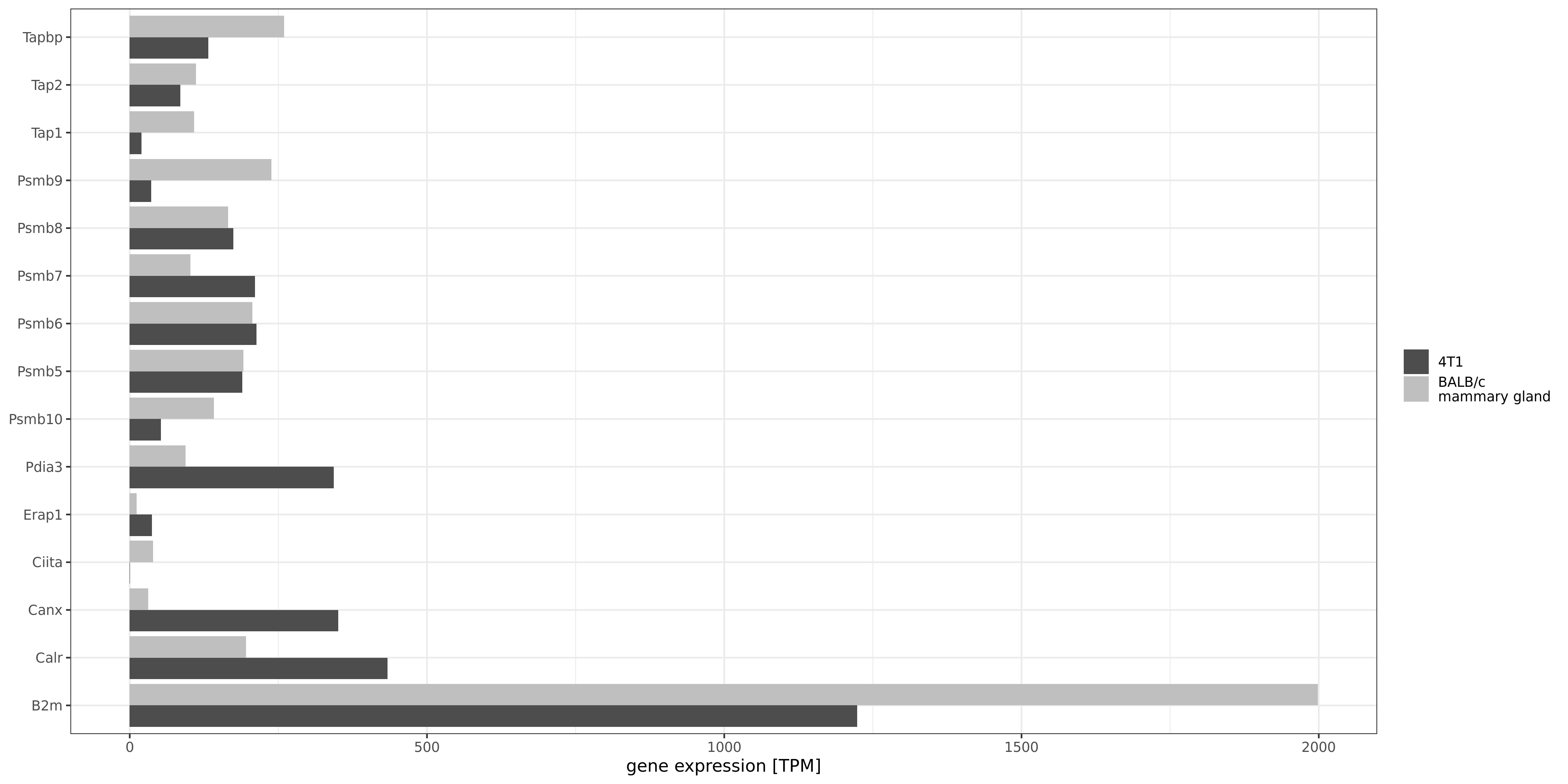

Supplement: Figure S8 — Gene expression of members of the MHC class I and II antigen presenting pathway in 4T1 and BALB/c mammary gland. [file Image_8.TIFF]
